# Inversion time calculations have varying impact on short, intermediate and long MOLLI T1 values: implications for studies using T1-mapping sequences

**DOI:** 10.1186/1532-429X-17-S1-P23

**Published:** 2015-02-03

**Authors:** Stefan K Piechnik, Vanessa M Ferreira, Andreas Greiser, Bruce S Spottiswoode, Matthew D Robson, Stefan Neubauer

**Affiliations:** 1 Oxford University, Oxford, UK; 2 Siemens Healthcare, Erlangen, Germany

## Background

T1-mapping has great diagnostic potential based on tight normal ranges and excellent sensitivity to disease. Numerically, T1 calculation hinges on the inversion time (TI) from the inversion preparation to the centre of k-space for each T1-weighted image. In the original ShMOLLI sequence (*Piechnik JCMR 2010*, *12:69*) based on "Work In Progress" WIP561 MOLLI, the TI calculation was offset from centre of k-space by two SSFP echo spacings, and included ~10ms duration of the adiabatic inversion pulse itself. The impact on T1 values resulting from rectifying this since WIP448B and WIP780 warrants investigation to assure an accurate clinical interpretation of previously published normal ranges of MOLLI T1 values.

## Methods

We compared pre- and post-contrast T1 values in multiple organs using original ShMOLLI (WIP561) and ShMOLLI (WIP448C) prototype sequences at 1.5T (Siemens Avanto MR system; software version syngo MR B17A) in 15 patients (7 males, 58±16 years-old, HR 64±11ms). A single mid-ventricular short-axis view per subject was used to acquire a pair of T1-maps using both versions; post-contrast T1-maps were acquired in threes (e.g. ShMOLLI WIP 561, 448, 561), averaging the 1^st^ and 3^rd^ measurements before comparing with the 2^nd^ to compensate for the time trend post gadolinium administration. Matched regions of interests were prescribed manually in left ventricular (LV) myocardium, LV and RV blood pool, spleen, liver parenchyma and visceral fat for comparison of the T1 values.

## Results

**Figure 1:**
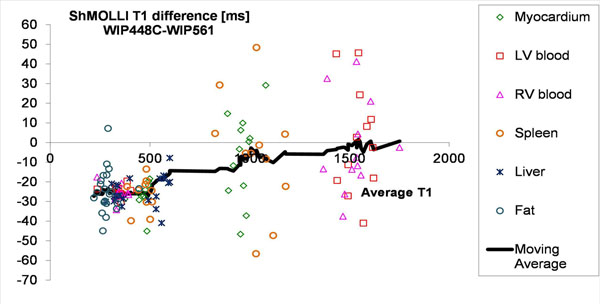
Bland-Altman plot displaying differences in T1 estimation over a wide range of *in-vivo* T1 values using original ShMOLLI (Siemens WIP 561) and the more recent ShMOLLI variant (Siemens WIP 448C).

131 paired T1 measurements were obtained for comparisons of native and post-contrast T1 values in different tissues (Figure 1). The novel ShMOLLI variant (WIP448C) produced lower T1 estimates compared to original ShMOLLI (WIP561). This is especially pronounced for the short T1 range, both in the pre-contrast environment (e.g. native liver and visceral fat T1) and all post-contrast T1 estimates (ΔT1= -25±8ms). The impact was less pronounced for the intermediate T1 range (i.e. native LV myocardium and spleen; -5±26ms) and nil for long T1 (LV and RV blood; -0.5±25ms). Specifically, native myocardial T1 showed trend level significance towards lower values (-7±21ms, p=0.094) measured using novel ShMOLLI WIP448C.

## Conclusions

There are quantitative differences in ShMOLLI-T1 estimation between WIP 561 and 448C versions due to inversion time calculations, which likely extends to other MOLLI variants. The effect is most pronounced on short T1 ranges (e.g. post-contrast T1) and may require correction of ~25ms to obtain equivalent values between WIP packages. The effect on intermediate and long T1 ranges (e.g. native myocardial and blood T1) is much less pronounced, but requires further investigation to establish corrections to previously established norms and thresholds for detecting disease. We suggest that studies employing T1-mapping sequences clearly publish the WIP number and software version to enable comparison of results to any published literature.

## Funding

The research was supported by the National Institute for Health Research (NIHR) Oxford Biomedical Research Centre based at The Oxford University Hospitals Trust at the University of Oxford.

